# Stem Cells and Engineered Scaffolds for Regenerative Wound Healing

**DOI:** 10.3390/bioengineering5010023

**Published:** 2018-03-09

**Authors:** Biraja C. Dash, Zhenzhen Xu, Lawrence Lin, Andrew Koo, Sifon Ndon, Francois Berthiaume, Alan Dardik, Henry Hsia

**Affiliations:** 1 Department of Surgery (Plastic), Yale School of Medicine, New Haven, CT 06510, USA; zhenzhen.xu@yale.edu (Z.X.); andrew.koo@yale.edu (A.K.); sifon.ndon@yale.edu (S.N.); 2 Department of Public Health Studies, Johns Hopkins University, Baltimore, MD 21218, USA; llin31@jhu.edu; 3 Department of Biomedical Engineering, Rutgers University, The State University New Jersey, Piscataway, NJ 08901, USA; fberthia@soe.rutgers.edu; 4 Department of Surgery (Vascular), Yale School of Medicine, New Haven, CT 06510, USA; alan.dardik@yale.edu

**Keywords:** chronic wound, scaffold, natural polymer, synthetic polymer, stem cell, surface modification

## Abstract

The normal wound healing process involves a well-organized cascade of biological pathways and any failure in this process leads to wounds becoming chronic. Non-healing wounds are a burden on healthcare systems and set to increase with aging population and growing incidences of obesity and diabetes. Stem cell-based therapies have the potential to heal chronic wounds but have so far seen little success in the clinic. Current research has been focused on using polymeric biomaterial systems that can act as a niche for these stem cells to improve their survival and paracrine activity that would eventually promote wound healing. Furthermore, different modification strategies have been developed to improve stem cell survival and differentiation, ultimately promoting regenerative wound healing. This review focuses on advanced polymeric scaffolds that have been used to deliver stem cells and have been tested for their efficiency in preclinical animal models of wounds.

## 1. Introduction

Wounds result from a disruption of the normal architecture of the skin [1]. Wound healing involves the coordination of many distinct but spatiotemporally overlapping physiological processes aimed at restoring the structural and functional integrity of the skin as a barrier to external stressors. This includes hemostasis, inflammation, cellular proliferation, angiogenesis, extracellular matrix (ECM) deposition, scar formation, and remodeling. These processes are highly regulated by the secretion of various cytokines, chemokines, and growth factors [2,3,4,5,6,7]. Disruptions in signaling during any of these stages can lead to chronic wound formation. 

Blood vessels are damaged during wound formation, initiating the clotting cascade. The resulting fibrin matrix serves as a site for cellular attachment, as well as a reservoir for cytokines and growth factors needed during latter phases of healing [2,4]. Platelets release various pro-inflammatory cytokines including platelet derived growth factor (PDGF), transforming growth factor beta (TGFβ), and fibroblast growth factor (FGF) [6]. Polymorphonuclear cells, followed by macrophages, migrate from the intravascular space to the site of injury. Together, these inflammatory cells phagocytose bacteria and cellular debris while macrophages also release matrix metalloproteinases (MMPs) that degrade ECM. They also secrete cytokines including interleukin 1 (IL-1), interleukin 6 (IL-6), vascular endothelial growth factor (VEGF), and tumor necrosis factor alpha (TNFα), which promote further cellular recruitment and proliferation of keratinocytes and fibroblasts [2,4,6]. 

Fibroblasts migrate to the wound matrix and release collagen, fibronectin, and proteoglycans, replacing the initial fibrin matrix with new ECM. In response to the hypoxic wound environment, they too release cytokines, including fibroblast growth factor (FGF), hepatocyte growth factor (HGF), TGFβ, EGF, and VEGF. In conjunction with signaling factors released during earlier stages, these promote the formation of new blood vessels, a critical component of acute wound healing [7]. Re-epithelialization then occurs as epithelial cells migrate from the periphery to the center of the wound. In full thickness wounds, stem cells from surrounding hair follicles and the interfollicular epidermis also migrate to the wound, contributing to the process of re-epithelialization [8]. Later phases of wound healing are characterized by further ECM deposition, wound contraction and scar formation, and tissue remodeling. 

Progression from an acute to chronic wound results from various local or systemic factors that alter the wound microenvironment. Local factors (e.g., tissue ischemia, infection, the presence of necrotic tissue, and ionizing radiation), as well as systemic factors (e.g., diabetes, smoking, and malnutrition) impair wound healing through prolonged inflammation, reduced angiogenesis, and a decrease in growth factor signaling. Additionally, chronic wounds have been found to have increased expression of MMPs causing excessive wound matrix degradation and prolonged healing, as is the case in diabetic patients [9]. 

Treating wounds and their associated complications places a large financial burden on the healthcare system, which exceeded $25 billion annually in the US by 2009 [10]. Traditional wound care involves infection control, debridement, selecting appropriate dressings to maintain a favorable wound healing environment, and addressing the underlying cause, such as ischemia or diabetes. However, the efficacy of current treatment modalities is limited for complex wounds. Recent advancements in the field of regenerative medicine, such as growth factor delivery and cell therapy, provides additional therapeutic options to potentially facilitate faster wound healing and restoration of normal skin architecture [7,11,12]. 

Among cell-based therapy, the use of stem cells for their regenerative wound healing potential is gaining widespread recognition. Many types of adult stem cells have been used in clinical trials for wound healing, but none has been approved yet [12,13,14,15]. Major limitations to clinical translation of stem cell-based therapies include stem cell immunogenicity and their reduced survival and paracrine activity in vivo. Polymeric biomaterial systems, especially biopolymer-based and/or surface modified scaffolds have been reported to be immunomodulatory by modulating innate immune response from inflammatory M1 phenotype towards a regenerative M2 phenotype. Furthermore, these scaffolds have shown promotion of wound healing by remodeling the nondermal tissue and recruiting endogenous stem cells to the chronic wound site [7,11,16,17]. These polymeric scaffolds have been developed to reduce the immunogenic effect of these stem cells and protect and improve their regenerative capacity within the chronic wound environment. 

This review focuses primarily on various polymeric biomaterial-based delivery vehicles that have been developed to date for stem cell delivery to wounds. Our goal is to discuss stem cell delivery systems that have been specifically tested in animal models of wounds. Here, we first describe various stem cells that have been utilized for wound healing applications and discuss their delivery by natural and synthetic polymer-based scaffolds to acute and chronic wounds. Furthermore, we have a section on surface modification describing different modification strategies of these scaffolds to promote stem cell survival and wound healing. 

## 2. Stem Cells for Regenerative Wound Healing

Stem cells have self-renewal and multipotency abilities that may help in creating advanced tissue engineered skin substitutes. Among the main sources of cells that may be used to engineer such substitutes are adult stem cells, embryonic stem cells (ESCs), and induced pluripotent stem cells (iPSCs). These cells have the potential to secret paracrine factors, making them an attractive option for the treatment of acute and chronic wounds [18]. So far, adult stem cells, especially mesenchymal stem cells (MSCs), have been widely used for regenerative healing [12]. MSCs can self-renew and have the ability to differentiate into osteoblastic, adipocytic, and chondrocytic lineages. MSCs can be isolated from various tissues, including bone marrow, adipose tissue, umbilical cord blood, nerve tissue, and skin dermis. They modulate wound healing by secreting paracrine factors such as VEGF, stromal cell derived factor (SDF-1), epidermal growth factor (EGF), keratinocyte growth factor (KGF), insulin like growth factor (IGF); enzymes such as matrix metalloproteinase-9 (MMP-9); and immunomodulatory cytokines including interferon-λ, TNF-α, IL-1α and IL-1β [18,19]. Some other adult stem cells that have been used for wound repair are hair follicle stem cells (HFSCs), epidermal stem cells, and unrestricted somatic stem cells (USSC) [20,21,22]. 

ESCs, derived from the inner cell mass of the blastocyst, have the ability to differentiate into any cell type, including skin cells [23,24,25], and have immense regenerative potential. Aside from ethical concerns and the substantial legal restrictions of the use of ESCs in any capacity, another major limitation of using ESC-derived cells for regenerative wound healing is their allogeneic and immunogenic nature [26]. 

iPSCs are the newest class of pluripotent stem cells first developed in 2006 by Takahashi and Yamanaka [27]. Like ESCs, iPSCs can differentiate into all types of cells, but they can be derived from adult somatic cells of the body through the induced expression of four factors: Oct-3/4, Sox2, c-Myc, and KLF4 [27]. This revolutionary technology allows for generation of autologous pluripotent stem cell populations, thereby avoiding immunogenicity and the ethical issues associated with human ESCs [28]. iPSCs have already been used to derive skin cells—including folliculogenic human epithelial stem cells, fibroblasts, and keratinocytes—to engineer skin substitutes [29,30,31,32]. The use and application of stem cells in regenerative wound healing have been extensively reviewed in several recent clinical and scientific publications [8,12,14,15].

## 3. Polymer-Based Biomaterials for Stem Cell Delivery for Regenerative Wound Healing

Over the last few decades, stem cell delivery using polymer based biomaterials has been the subject of intense investigation [33,34]. These biomaterials can be fabricated using natural polymers such as hyaluronan, chitosan and alginate, collagen, elastin, fibrin, and silk [35,36] and genetically engineered peptides [37,38] or synthetic polymers including poly(lactic-*co*-glycolic) acid, polyanhydrides, polyethylene glycol, and others. These different polymers have specific physio-chemical characteristics and functional groups that allow for precise control of the creation of biomaterials with desired properties for a wide range of cell therapy applications. However, it is to be noted that the most important features critical to the success of any polymer are bioactivity, biodegradability, and biocompatibility [35].

### 3.1. Natural Polymer-Based Biomaterials 

Natural polymers such as collagen, fibrinogen, tropoelastin, hyaluronic acid, glycosaminoglycans, etc., are found in the extracellular matrix. Scaffolds fabricated using these polymers are biocompatible, bioactive, assist in cell attachment to cell surface receptors, and provide a niche to control cell function [34]. Some other natural polymers that have been used in designing biomaterials include plant, insect, or animal derived components, such as cellulose, chitosan, silk fibroin, etc., and have cell attachment sites and other properties to provide favorable microenvironments for stem cell delivery. Refer to Table 1 for further information on various biopolymers and their use in wound healing applications. Some of the disadvantages of these polymers include difficulty of sterilization and purification, high lot-to-lot variability, and a high potential for pathogen contamination during the isolation process. Additionally, there is limited ability to control physio-chemical properties and degradation rates of scaffolds made using these materials [39,40,41]. 

Collagen, one of the major ECM-based proteins, has been used widely to fabricate scaffolds for stem cell delivery. Bone marrow derived MSCs (BM-MSCs), adipose derived MSCs (ADSCs), and other adult stem cells have been delivered using collagen scaffolds. O’Loughlin et al. fabricated a collagen scaffold to deliver BM-MSCs to a diabetic rabbit ear ulcer wound model. Topical application of the MSC-collagen treatment in a dose-dependent fashion significantly increased wound closure rates compared to that of untreated groups. Higher cell doses yielded better wound closure and faster rates of wound healing. The wound closure was associated with increased angiogenesis in the MSC-collagen groups [63]. A similar study by Kim et al. showed delivery of BM-MSCs in collagen gel scaffolds to full thickness skin wounds of rats. The wound size was significantly reduced in rats treated with MSC-collage scaffold compared to collagen alone on day 7. There was no difference by day 14, suggesting that MSCs appear to accelerate early wound healing. MSC-collagen treated mice also had significantly more expression of VEGF and more intense early expression of MMP-9 compared to collagen treatment without MSCs, indicating a possible mechanism of MSCs’ effects on wound healing [64]. Similarly, other stem cells such as ADSCs and Leucine Rich Repeat Containing G Protein-Coupled Receptor 6 (LGR6+) epithelial stem cells have been delivered to wounds using collagen scaffolds [65,66]. 

Fibrin, another ECM-based protein, has been shown to form gels by reacting with thrombin. Although not investigated as extensively as collagen, fibrin has also been studied as a potential scaffold material for the delivery of stem cells. Ozpur et al., described a method of creating artificial skin tissue by using fibrin gels seeded with keratinocytes and ADSCs. This skin substitute promoted healing by complete re-epithelialization of the wound with no contraction and increased vessel density and collage deposition [49]. Another group, Falanga et al., also constructed gels to carry MSCs. In in vivo experiments, diabetic mice with full thickness wounds demonstrated accelerated healing with topical application of MSC and fibrin compared to fibrin alone. In human patients, acute wounds treated with fibrin gel demonstrated healing and resurfacing. Treatment of human chronic wounds with fibrin and MSC resulted in increased wound closure in wounds that were previously refractory to other therapies. A significant correlation was found between the number of MSCs applied and the subsequent decrease in size of the chronic wounds [67]. 

Another important ECM bio-macromolecule is elastin, which has been used for various tissue engineering applications, including in the form of tropoelastin [68,69]. In the work of Machula et al., human ADSCs were seeded on recombinant tropoelastin-based electrospun scaffolds. The ADSC-seeded scaffold formed a stable structure with high biocompatibility. The scaffold demonstrated rapid cell proliferation with deposition of new ECM. Experimentation with the seeded scaffold in vivo with a murine excisional wound model revealed greater would closure as well as greater epithelial thickness [48].

Silk protein is a natural fibrous protein that has been used for various tissue engineering applications. In a study by Navone et al., AD-MSCs were obtained from human lipoaspirates and tested for the ability to differentiate both before and after seeding on silk fibroin (SF) scaffolds. Sheets of the silk fibroin scaffold were prepared through electrospinning and were loaded with MSCs to generate cellularized scaffolds (AD-MSCs-SF). The SF scaffolds allowed for growth of AD-MSCs as well as retention of differentiation capacity. Evaluation of both the cellularized and decellularized scaffolds in diabetic mice revealed improved tissue regeneration and enhanced expression of angiogenesis-related genes, corroborated by in vitro analysis. The ability of the scaffolds to affect migration of human umbilical vein endothelial cells (HUVEC), keratinocytes, and dermal fibroblasts was also observed, with enhanced HUVEC migration and release of angiogenic factors. Interestingly, the decellularized scaffold demonstrated comparable efficacy to the cellularized scaffold, showing potential for future application in wound care [52]. 

Hyaluronic acid (HA) or hyaluronan is another major component of ECM. It contains sites for cell adhesion and has been widely used for surface modification of scaffolds and/or hydrogels to enhance cell survival and proliferation. Recently Gerecht et al. used acrylated HA (AHA) to form a smart hydrogel containing cell adhesive peptide and MMP-sensitive peptide cross-linker. The AHA hydrogel was used to deliver early vascular cells derived from human iPSC (hiPSC). The vascularized scaffold showed enhanced angiogenesis and wound closure in diabetic immunodeficient mice [70]. 

Chitosan, xanthan, and carboxymethylcellulose are polysaccharides that have been used to culture stem cells. More information regarding other polysaccharide-based polymers can be found in Table 1. Bellini et al. formulated a 3D membrane structure comprised of chitosan and xanthan, which showed adherence of MSCs and demonstrated improved wound healing in rat models of wound healing [58]. Similarly, Rodrigues et al. extracted rat ADSCs from adipose tissue and combined them with a sodium carboxymethylcellulose (CMC) scaffold. Evaluation of CMC toxicity in vitro revealed no significant increase in lactate dehydrogenase activity that would suggest increased cell death. The results of in vivo testing of CMC associated with ADSCs in a rat wound model revealed increased cell proliferation rates of the granulation tissue as well as greater epithelium thickness when compared to untreated rats. However, there was no increase in collagen fibers, nor were there changes in the speed of wound closure [71].

### 3.2. Synthetic Polymer-Based Biomaterials

Synthetic polymers are an alternative to natural materials and have well-defined and tunable chemical and mechanical properties. They present some advantages over natural polymer based biomaterials including their ability to be fabricated to have specific degradation rates. Thus a wide array of synthetic polymers has been explored for almost all tissue engineering applications [36]. Refer to Table 2 for a list of various synthetic polymers and their application in wound healing.

Chen et al., reported a hydrogel of poly *N*-isopropylacrylamide (PNIPAM) and poly(amidoamine) (PAA). The PNIPAM-PAA hydrogel was used to deliver BM-MSCs to ulcers in a diabetic mice. The hydrogel alone and with BM-MSCs showed reduction of M1 inflammatory macrophages. BM-MSC-laden hydrogels induced rapid healing of diabetic ulcer by increasing angiogenesis, granulation tissue formation, ECM secretion, wound contraction, and re-epithelialization [77]. 

Poly(vinyl alcohol) (PVA) is a synthetic polymer that has been widely used for drug delivery and tissue engineering application [79]. In one study, a crosslinked PVA membrane was used for the delivery of Wharton’s jelly MSCs (WJ-MSCs) [80]. The PVA membrane supported cell adhesion and proliferation. WJ-MSCs delivered using PVA membranes showed improved healing in a canine wound model. 

Poly-l-lactic acid (PLLA) and poly lactic-*co*-glycolic acid (PLGA) are biodegradable synthetic polymers that have been used to deliver stem cells to wounds [72,76]. Kim et al. studied the effect of umbilical cord derived endothelial progenitor cells (CB-EPCs) in a PLLA scaffold on wound healing in vivo. These biodegradable PLLA scaffolds were modified with Arg–Gly–Asp (RGD) peptide to promote cell adhesion. The RGD-*g*-PLLA scaffold was shown to successfully support the growth and endothelial functions of these CB-EPCs in vitro. In murine dermal wound models, the CB-EPC-seeded scaffold promoted vascular regeneration and was superior to conventional intradermal CB-EPC injection treatments in terms of localization and survival/retention of EPCs, as well as neovascularization at the wound bed. Similarly, another work by Yang et al. studied the effect of scaffolds seeded with VEGF-enhanced stem cells on wound healing in mice. Scaffolds made using PLGA and PLLA were used to deliver VEGF enhanced human MSCs. Subcutaneous implantation of the scaffold plus enhanced human MSCs demonstrated increased blood vessel migration into the constructs compared to implantation of stem cells transfected by a control plasmid and those transfected with VEGF using a commercial reagent [76]. 

Some other biodegradable synthetic polymers that have been used for regenerative applications are polycaprolactone (PCL) and polyethyleneglycol (PEG). Bahrami et al., fabricated an electrospun nanofibrous scaffold using PCL to deliver USSCs. The scaffold, which was modified with laminin, showed good cell viability, and in vivo study showed increased wound closure and a thin epidermis with recovered skin appendages in the dermal layer [22]. Similarly, Gu et al., fabricated a scaffold using PCL and poloxamer (PLCL/P123) to deliver rat ADSCs. The ADSC-seeded scaffolds were evaluated in vivo through a rat skin tissue injury model, producing the highest percentages of wound closure when compared to the PLCL/P123 scaffold alone or with a control petrolatum gauze, indicating enhanced wound healing. Rats treated with the ADSC-seeded scaffolds also exhibited greater microvessel density. The regenerated skin demonstrated a thick and integrated epidermal structure [78].

Geesala et al. produced a semi-inter penetrating network porous 3D scaffold using PEG-polyurethane (PEG-PU) and PEG-dimethylether (PEGDME). The scaffold was also shown to be thermostable, biodegradable, cytocompatible, and protective against oxidative stress. The scaffold loaded with BM-MSCs promoted healing with an increase in fibroblast proliferation, collagen deposition, and anti-oxidant enzyme activities. Furthermore, it reduced proinflammatory cytokines IL-1b, 6, 8, and TNF-α and upregulated anti-inflammatory cytokines IL-10 and 13. Increased engraftment was seen by expression of Sca-1+Lin-CD90+CD133+. Improved neovascularization was also evident, with increased expression of PECAM, VEGFR3, neuropilin 2, and Tie2 [73]. In the work of Dong et al., an injectable hydrogel was prepared using a thermoresponsive hyperbranched copolymer of poly(ethylene glycol) methyl ether methacrylate-*co*-2-(2-methoxyethoxy) ethyl methacrylate-*co*-poly(ethylene glycol) diacrylate (PEGMEMA-MEO_2_MA-PEGDA). The hydrogel supported cell viability and proliferation of rat ADSCs. In vivo testing in a rat dorsal full-thickness wound model revealed that the hydrogel system improved angiogenesis [81]. A modification of the PEGMEMA-MEO_2_MA–PEGDA hydrogel with HA showed a marked increase in secretion of growth factors over a 7-day period [82]. Lee et al. investigated wound healing in diabetic mice using muscle-derived stem cells (MDSCs) and a thermosensitive hydrogel made of the triblock co-polymer PEG-PLGA-PEG. In vivo*,* wounds of diabetic mice treated with MDSCs in the PEG-PLGA-PEG hydrogel showed increased epithelium migration, collagen deposition, engraftment of MDSCs, and enhanced wound closure rates within the first ten days after treatment [74].

Though these synthetic polymers allow for engineering of biomaterials with tunable properties (e.g., chemical and mechanical properties and degradation rates), disadvantages for choosing such materials include poor bioactivity due to a lack of cell attachment sites and acidic byproducts that can trigger an immune response [39,40,41,83]. It is thus critical to modify synthetic materials with biological or chemical entities to achieve an appropriate cellular response. 

## 4. Modifications to Biomaterials

The interaction between cells and the biomaterial surface dictates the survival and function of stem cells within scaffolds. Surface modification of a biomaterial by incorporating suitable biological and chemical cues can ultimately control stem cell activity by manipulating the signal transduction pathways in stem cells after its attachment on the surface [39,40]. Surface modification of scaffolds is therefore an important aspect in tissue engineering in order to control cellular behavior [83]. This section will discuss different methods that have been utilized to modify scaffolds for stem cell delivery for wound healing (Table 3). 

Modifications to collagen scaffolds have been made to replicate the ECM environment of wounds by addition of biomacromolecules such as glycosaminoglycans (GAGs) or laminin. Liu et al. (2008) created collagen–GAG scaffolds to study wound healing in porcine models. In in vivo partial thickness burn wounds, treatment with MSC-seeded scaffolds showed improved healing and keratinization, less wound contraction, and more vascularization compared to scaffold alone or no treatment. Labeled MSCs were also detected in the epidermal and dermal components of the wound bed, indicating that they had migrated from the scaffold and were integrated into the neoepidermis and neodermis [84]. In another study, Assi et al. used a collagen scaffold to deliver BM-MSCs to treat diabetic ulcers [51]. Laminin was added for functionalization of the scaffold. The functionalized collagen scaffold promoted survival of MSCs and increased secretion of VEGF. Laminin further enhanced the healing effect of the scaffold. 

Catanzano et al., used an alginate (ALG)-HA hydrogel for dermal regeneration [85]. HA of 10–20% of the ALG weight was incorporated in a physically crosslinked ALG hydrogel. The integration of HA showed therapeutic efficacy of the hydrogel by significantly promoting gap closure in an in vitro study and by maintaining cell survival of ADSCs and keratinocytes. In an in vivo rat excisional wound model, the use of the alginate-HA hydrogel promoted wound closure compared to ALG alone. In another work, Schmitt et al. synthesized calcium alginate gels by both internal and external crosslinking, and incorporated PEG 300,000 and HA for cell adhesion [56]. They observed cell survival within the gel for 6 weeks and secretion of paracrine factors such as VEGF and bFGF. They theorized that the gel can be used for wound healing applications. In another study by Cerqueira et al., human ADSCs and microvascular endothelial cells (MECs) were obtained from human adipose tissue [62]. The cells were cultured and combined with gellan gum–hylauronic acid (GG–HA) hydrogels. The hydrogels demonstrated sponge-like properties with composition and physical properties similar to the ECM of the skin. The GG-HA hydrogels promoted neovascularization that was further augmented by the inclusion of MECs. The GG-HA hydrogels absorbed early inflammatory cell infiltrates and led to the formulation of granulation tissue, with greater epidermal thickness observed in mice implanted with hydrogels than those without. Hydrogel degradation was observed, along with improvements in wound closure, re-epithelialization, and matrix remodeling. 

Silk protein sericin has widely been used for various biomedical application [86]. Recently, sericin has been used to modify collagen scaffolds for the culture of stem cells. In one of the studies, the researchers showed that the addition of sericin yielded an increased rate of proliferation of ADSCs seeded within collagen gel, compared to a pure collagen control. Sericin also stimulated the overexpression PPARγ2 [87]. In another work by Kim et al., MSCs were seeded on a collagen scaffold modified by silkworm gland hydrolysate (SSGH). The scaffold demonstrated cytocompatability when evaluated in vitro with MSCs. The SSGH/collagen scaffold also demonstrated antioxidant properties, mitigating cell damage induced by oxidative stress. Experimentation with the SSGH/collagen scaffold in a mouse full-thickness excisional wound model revealed accelerated wound re-epithelialization and decreased wound-healing time while maintaining optimal tissue hydration. The observations suggest that the SSGH/collagen scaffold may stimulate growth factor production in healthy cells found in the wound site [88]. In another work, Bhowmick et al. also generated electrospun nanofibrous scaffolds with gelatin, HA, chondroitin sulfate, and sericin. Sericin modified scaffold promoted cell viability and proliferation of human fibroblast cells, keratinocytes, and MSCs. Further co-culture of keratinocytes and hMSCs showed differentiation of hMSCs towards the epithelial lineage. The scaffold thus is a promising treatment for dermal regeneration [46]. 

In another work, Fibrin fragment E (FbnE) was used for the modification of an alginate scaffold and used for culturing cord blood epidermal progenitor cells (CB-EPCs). FbnE was shown to promote adhesion and endothelial differentiation of CB-EPCs in vitro. In vivo, co-administration of the FbnE-enriched scaffold with CB-EPCs accelerated wound closure and vascularization compared to administration of the FbnE scaffold alone [50]. 

In a work by Natesan et al., PEGylated fibrin was used along with collagen to fabricate a bilayer hydrogel to deliver ADSCs. The authors found that the ADSCs successfully proliferated and differentiated in the bilayer hydrogel in vitro. They formed dense tubular microvascular networks in the PEGylated fibrin-based hydrogel. The bilayer hydrogel decreased wound contraction and increased wound closure time compared to the saline-treated control. In addition, rats treated with the ADSCs–bilayer hydrogel showed a significant increase in granulation tissue formation, re-epithelialization of wound margins, and better progression of the epithelial margin toward the center of the wound [75]. Similarly, Xu et al. utilized a gelatin modified PEG hydrogel. MSCs containing gelatin/PEG hydrogel were applied to a full thickness wound model in rats. By day 7, the seeded gels showed significantly accelerated wound closure and reepithelialization, and improved angiogenesis and granulation tissue formation [89].

## 5. Conclusions and Future Directions

**Figure 1:**
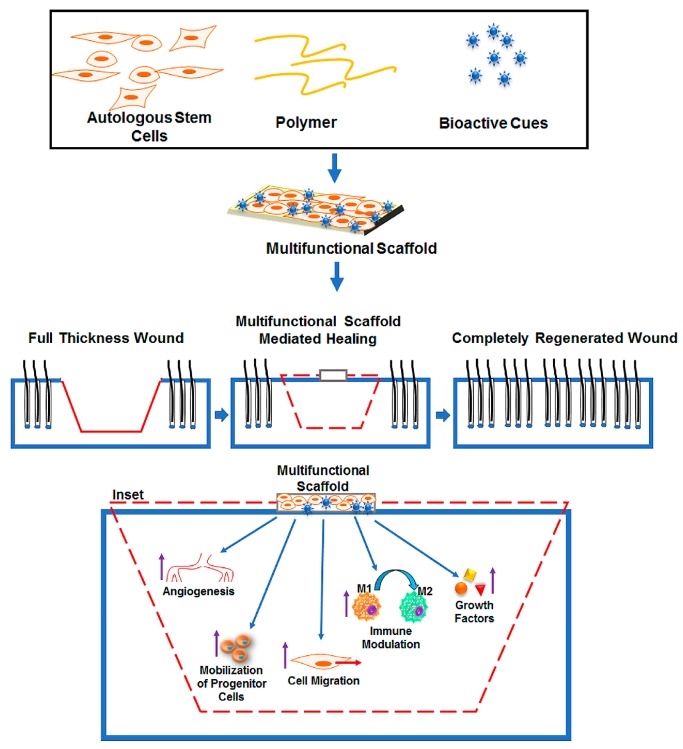
Schematic showing development of a personalized regenerative treatment modality for chronic wound patients by fabricating a multifunctional scaffold system using autologous stem cells, different bioactive cues, and polymer of interest. The scaffold when implanted into a full thickness wound would promote regenerative healing (scar-free) by providing a vascular bed, modulating inflammatory M1 macrophages to a pro-healing M2 macrophages, inducing movement of progenitor cells and increased migration of fibroblast and keratinocytes.

Advancements in the field of stem cell biology and biomaterials have created exciting opportunities for wound healing therapy. Polymeric biomaterials can now be rationally designed to provide a niche for the stem cells to survive and function. The purpose of this review was to detail some of the most commonly used biomaterial scaffolds for transplantation of stem cells for wound healing applications. It sought to detail the types of materials available and highlight their unique properties with respect to stem cell delivery with an emphasis on how they perform in vivo in preclinical models of wounds. We believe this will help readers choose a material that would best suit their specific wound healing needs. This review also touched on how modifications to biomaterials can play a large role in further promoting stem cell function and regenerative wound healing. Other considerations when designing such scaffolds including the method of fabrication and composite scaffolds (e.g., blending of two or more different polymers to fabricate composite scaffolds to further achieve additional desired properties and benefits) are beyond the scope of this review. Ultimately there are a number of significant challenges researchers must overcome before a stem cell and biomaterial-based option for wound healing therapy can be used in the clinical setting. In addition to overcoming the difficulty of obtaining a renewable source of stem cells in large quantities, major advances in both the understanding of the local cues necessary for stem cell survival and function and development of biomaterials necessary to promote these functions is key. These polymer-based biomaterials should be tested in a variety of in vivo and in vitro studies prior to their application with the stem cells on a clinical level. The future lies in utilizing high throughput arrays to test the functionality of new biomaterials for their usage in stem cell delivery in short periods of time while wasting few materials, ultimately allowing for a more rapidly developed end product. Further progress in this field involves utilizing a hybrid approach to produce personalized tissue engineered constructs by using patient specific cells, biomimetic matrices (e.g., collagen, gelatin, chitosan, Fibrin etc.) and bioactive stimuli (e.g., Fibrin, laminin, silk protein, GAG etc.) to promote a regenerative healing (Figure 1). Furthermore, multiple cell types may also be employed with spatial control to generate skin tissues ex vivo, along with the requisite vascular supply. These advancements will lead to more clinical trials and finally allow the translation of stem cell therapy-based regenerative wound healing from bench-side research into bedside treatments for patients.

## Figures and Tables

**Table 1:** Biological polymers for wound healing application.

Biological Polymer	Structure	Selective Wound Healing Application	Important Findings	Reference
Collagen	A fibrous triple helical protein. Collagen type I, a major subtype consists of two alpha 1 units and one alpha 2	3D scaffold Hydrogel Composite material	MSCs within the scaffolds greatly ameliorated the quality of regenerated skin, reduced collagen deposition. Enhanced reepithelization, increased neo-angiogenesis, and promoted a greater return of hair follicles and sebaceous glands. The mechanisms involved in these beneficial effects were likely related to the ability of MSCs to release paracrine factors modulating the wound healing response. Self-assembled ASC spheroids on chitosan-hyaluronan membranes expressed more cytokine genes (fibroblast growth factor 1, vascular endothelial growth factor, and chemokine [C-C motif] ligand 2) as well as migration-associated genes (chemokine) [C-X-C motif] receptor type 4 and matrix metalloprotease 1. Spheroids combined with the use of biomaterials can enhance skin wound healing and more capillary formation. This study shows the potential use of biomaterial-derived 3D MSC spheroids in wound treatment. Increased the recruitment of provascular circulating bone marrow-derived mesenchymal progenitor cells in vivo. Significant increase in BM-MPC migration, proliferation, and tubulization when exposed to hydrogel-seeded ASC conditioned medium. BM-MPC expression of genes related to cell stemness and angiogenesis was also significantly increased following exposure to hydrogel-seeded ASC conditioned medium. ASC-seeded hydrogels improve both progenitor cell recruitment and functionality to effect greater neovascularization.	[42,43,44]
Gelatin	A hydrolytic byproduct of collagen	Microgel Composite material; nanofibers; electrospun scaffold	Biodegradable gelatin microgels (GMs), as 3D micro-scaffolds, could provide suitable microenvironment for stem cell proliferation. GMs could greatly improve growth factor secretion from hASCs and might be an enhanced strategy to promote hASCs-assisted wound healing. GMs could protect hASCs in the micro-niches and exhibit excellent injectability through syringe without obvious cell damages. hASCs delivered via GMs assisted injection could retain hASCs in situ and improve cell survival in wounds. Besides, hASCs retention improved by GMs could enhance secretion of positive tissue growth factors for wound healing, indicating an advanced injection strategy superior to free cell suspension injection GMs assisted cell delivery could accelerate wound healing by constant modulation of local growth factor expression to enhance regeneration and vascularization Treated wounds closed much faster, with increased re-epithelialization, collagen formation, and angiogenesis in vivo. USCs could secrete VEGF and TGF-β1. USC-conditioned medium enhanced the migration, proliferation, and tube formation of endothelial cells in vitro The composite nanofibrous scaffold was found to be biocompatible. Electrospun scaffold containing sericin promoted epithelial differentiation of hMSC.	[45,46,47]
Elastin	An elastic protein made up of water soluble tropoelastin	Electrospun scaffold	Electrospun tropoelastin membranes form stable structures that retain their integrity and strength in tissue culture medium. ADSCs rapidly proliferate on the scaffold and secrete an ECM that eventually covers the entire scaffold in vitro. The populated scaffold is well tolerated in a murine excisional wound model. Wounds treated with ADSC-populated tropoelastin scaffolds showed greater rate of closure and restoration of normal epithelium.	[48]
Fibrin	A fibrous non-globular protein produced by the cleavage of fibrinogen.	Gel Composite material	Enhanced wound healing for the scaffold containing ADSC and keratinocyte. Total epithelialization and higher collagen deposition and higher vascularization. Co-administration of FbnE enriched scaffold (SM) with CB-EPC accelerated wound closure and vascularization compared to FbnE scaffold alone. No differences in number of pericytes and myofibroblasts seen. No comparison to Integra treated mice as all died before study was complete.	[49,50]
Laminin	A heterotrimeric glycoprotein and binds to ECM proteins and cell membranes	Surface modification	The biomimetic collagen scaffold increases VEGF secretion from MSC in vitro. Activated MSC in collagen scaffolds increase wound healing in vivo. Activated MSCs increase wound healing in a splinted back wound model. Laminin improves wound healing efficiency. MSC delivered topically increase wound healing.	[51]
Silk protein	Extracted from cocoon of silk worms. It contains fibrous protein fibroin and water soluble sericin protein.	Scaffold Composite material Film	Scaffolds significantly improved tissue regeneration, reducing the wound area. Decellularized patches are almost as effective as cellularized patches in the treatment of diabetic wounds Scaffolds improve healing through the release of angiogenic and collagen deposition stimulating molecules. Silk Fibroin can be used a scaffold and is biocompatible, biodegradable and has excellent tensile strength The scaffold can support adipose derived stem cells for skin tissue engineering. Added Pectin and Glycerol can benefit scaffold by promoting protein conformation transition and biomaterial flexibility, respectively. BDNF-induced proliferation and migration of MSCs. BDNF stimulation affects the ability of MSCs to secrete IL-8, NGF, and MMP-9 and that this process depends largely on the Akt signaling pathway. The upregulation of NGF, IL-8, and MMP-9 in the BDNF-CM group contributed to angiogenesis. The BDNF-CM-modified materials also significantly accelerated wound healing. BDNF promotes angiogenesis and enhances the milieu-dependent endothelial differentiation of MSCs in ischemic ulcers.	[46,52,53,54]
Hyaluronic acid/Hyaluronan	An anionic nonsulfated glycosaminoglyacan that helps in cell migration and proliferation	Surface modification Composite material	Scaffolds seeded with VEGF165-modified rHFSCs, resulted in promotion of angiogenesis during wound healing and facilitation of vascularization in skin substitutes Increased VEGF165 level in the repair microenvironment improved vascularization ability. It is believed that HFSCs secrete a variety of cytokines to promote wound healing. Subcutaneous implantation showed that vascularization capacity of non-seeded SJS and ADM were greater than that of Co-CS-HA in subcutaneous wounds. ADSCs cultured on SIS secreted more VEGF compared to those seeded on ADM and Co-CS-HA ADSC-seeded scaffolds enhanced angiogenesis and wound healing rate compared to non-seeded scaffold in in vivo mouse models. ADSC-SIS and ADSC-ADM had greater microvessel densities than ADSC-*co*-CS-HA in vivo.	[20,55]
Alginate	An anionic polysachharide consisting of homopolymeric blocks of (1-4)-linked β-d-mannuronate (M) and C-5 epimer α-l-guluronate (G) residues.	Hydrogel Composite material	Humans MSCs remained viable for the duration of 6 weeks within the gels. Human VEGF and bFGF was found in quantifiable concentrations in cell culture supernatants of gels loaded with MSCs and incubated for a period of 6 weeks. Conditioned medium from mesenchymal stromal cells stimulates migration of dermal fibroblasts in scratch assays. Conditioned medium of mesenchymal stromal cells induces alterations in the expression of genes involved in wound healing. Encapsulated mesenchymal stromal cells retain stem cell characteristics and remain viable during long-term encapsulation. Encapsulated mesenchymal stromal cell-derived conditioned medium stimulates migration of dermal fibroblasts and induces alterations in the expression of genes involved in wound healing. Cells attached proliferated on the porous membrane. Accelerated wound healing.	[56,57]
Chitosan	A linear polysaccharide consisting of β-(1→4)-linked d-glucosamine and *N*-acetyl-d-glucosamine.	Membrane Scaffold Composite material	Conditioned medium from mesenchymal stromal cells stimulates migration of dermal fibroblasts in scratch assays. Conditioned medium of mesenchymal stromal cells induces alterations in the expression of genes involved in wound healing. Encapsulated mesenchymal stromal cells retain stem cell characteristics and remain viable during long-term encapsulation. Encapsulated mesenchymal stromal cell-derived conditioned medium stimulates migration of dermal fibroblasts and induces alterations in the expression of genes involved in wound healing. The scaffold was biocompatible UC-MSC differentiated to epidermis and positive for the epidermal markers cytokeratin 19 and involucrin at 14 days. The constructed epidermis substitutes helped rapid wound healing. Better cell adhesion, growth, and proliferation inside the modified scaffolds. Showed a good resilience and compliance with movement as a skin graft. All scaffolds, especially those with stem cells, exhibited pronounced effects on wound closure. The reconstructed skin in grafted groups demonstrated an intact epithelium with the formation of new hair follicles and sebaceous glands, which were reminiscent of the structures of normal skin.	[58,59,60]
Pullulan	A polysaccharide consisting of maltotriose units, connected by an α-1,6 glycosidic bond.	Hydrogel Composite material	Described above. Hydrogel seeding of ASCs resulted in the enhanced expression of multiple stemness and angiogenesis-related genes (Oct4, Vegf, Mcp-1, and Sdf-1) in vitro. ASCs seeded within hydrogel scaffolds showed minimal proliferation and maintained baseline levels of metabolic activity. Hydrogel delivery improved ASC survival in vivo. Resulted in accelerated wound closure and increased vascularity in splinted murine wounds.	[44,61]
Xanthan	A hetero-polysaccharide with main containing glucose units and side chain of trisaccharides	Membrane	Described above.	[58]
Gellan gum	A water soluble anionic polysaccharide with repeated tetrasaccharide units containing two d-glucose and one of each l-rhamnose and d-glucuronic acid	Hydrogel	The hydrogels absorbed early inflammatory cell infiltrate and led to formulation of granulation tissue in vivo. Improved wound closure, re-epithelialization, and matrix remodeling. Promoted superior neo-vascularization.	[62]

**Table 2:** Synthetic polymers for wound healing application.

Synthetic Polymer	Structure	Selective Wound Healing Application	Important Findings	Reference
Poly(l-lactic acid) (PLA or PLLA)	A biodegradable and thermoplastic polymer synthesized using monomers of lactic acid or lactide.	Scaffold	Scaffolds had a high porosity and a 50–75% increase in swelling, along with complete protein release in the presence of phosphate-buffered saline. Accelerated wound re-epithelialization in mouse model Maintained optimal hydration of the exposed tissues and decreased wound healing time in vivo.	[72]
Poly(ethylene glycol) (PEG)	A hydrophilic polymer synthesized by anionic ring-opening polymerization of ethylene oxide.	Scaffold Hydrogel Composite material	Polymer network/porous scaffold helps cells from oxidative stress. The implant showed fibroblast proliferation, collagen deposition, and anti-oxidant enzyme activity. Enhanced wound healing by enhanced engraftment and increased vascularization. Decreased proinflammatory cytokines and increased anti-inflammatory cytokines. Wounds treated with MDSC and PEG-PLGA-PEG showed enhanced wound closure rate, epithelium migration, and collagen deposition. There was increased engraftment of MDSCs into the wound bed compared to controls (MDSC treatment without hydrogel and MDSC with control dressing). In wounds, 25% MDSCs differentiated into fibroblasts, 10% into myofibroblasts, 10% into endothelial cells, and none into macrophages. Rat excision wounds treated with PEGylated fibrin-collagen bilayer hydrogels show decreased wound contraction over time. They show faster wound closure in comparison with control Within bilayer hydrogels, dsASCs proliferate, differentiate, maintain a spindle-shaped morphology in collagen, and develop tubular microvascular networks in PEGylated fibrin.	[73,74,75]
Poly(lactic-*co*-glycolic acid) (PLGA)	A biodegradable copolymer of glycolic and lactic acid.	Scaffold	Scaffolds seeded with VEGF-transfected stem cells led to increased blood vessel migration into the constructs compared to control cells or cells transfected with VEGF using a commercial reagent. There was increased endothelial cell density compared to the controls.	[74,76]
Polyurethane	A polymer synthesized by reacting poly-isocyanate and polyol. Contains urethane to join organic units.	Composite material	Described above.	[73]
Poly(*N*-isopropylacrylamide) (PNIPAM)	A thermoresponsive polymer synthesized using free radical polymerization of *N*-isopropylacrylamide.	Thermosensitive hydrogel	Hydrogel and BMSC combination therapy promoted wound contraction. The hydrogel inhibited chronic inflammation. Hydrogel and BMSCs combination therapy promoted the formation of granulation tissue. Hydrogel and BMSCs combination therapy promoted keratinocyte proliferation and differentiation. Hydrogel and BMSCs combination therapy improved the quality of wound healing.	[77]
Polycaprolactone (PCL)	A biodegradable polyester prepared by ring opening polymerization of ε-caprolactone.	Composite material	ADSCs differentiated into epidermal-like structures Observed higher microvessel density in rat skin tissue injury models. Improved healing was observed in vivo. ADSCs-PLCL/P123 scaffolds with the thickness of 150–250 μm match well with the epidermis layer (200–250 μm).	[47,78]
PAA-poly(amidoamine)	A dendrimer with repetitively branched subunits of amide and amine	Hydrogel	Described above.	[77]

**Table 3:** Surface modifications of scaffolds for wound healing application.

Biopolymers for Surface Modification	Effect on Stem Cells and Wound Healing
Glycosaminoglycan	Promoted MSC survival. Improved healing, keratinization and vascularization [84].
Laminin	Enhanced MSC survival and VEGF secretion. Promoted healing [51].
HA	Maintained cell survival of ADSC and keratinocytes and improved wound closure [85]. Enhanced cell adhesion and survival and secretion of paracrine factors such as VEGF and bFGF [56]. Enhanced neovascularization, wound closure, re-epithelialization, matrix remodeling and reduced inflammation [62].
Sericin/Silk derivative	Increased cell proliferation of ADSC and maintained adipogenicity of the cells by stimulating the expression of PPARγ2 [87]. Reduced oxidative stress in the cells, enhanced re-epithelialization and wound closure [88]. Promoted cell viability and proliferation of MSCs and keratinocytes and fibroblasts and helped differentiation of MSCs to epithelial lineage [46].
Fibrin	Fibrin fragment E promoted cell adhesion and differentiation of cord blood epidermal progenitor cells to endothelial cells and enhanced vascularization and wound closure [50]. PEGylated fibrin promoted cell proliferation of ADSCs and tubular microvascular formation in the scaffold and enhance wound closure, re-epithelialization [75].
